# Plasminogen Activator Inhibitors in Thrombosis: Structural Analysis and Potential Natural Inhibitors

**DOI:** 10.1021/acsomega.5c02926

**Published:** 2025-06-19

**Authors:** Roney V. dos Santos, Manoel Victor Frutuoso Barrionuevo, Mikael Rangel Fernandes Vieira, Ivan Mazoni, Ljubica Tasic

**Affiliations:** a School of Pharmaceutical Sciences, UNICAMP, Campinas, SP 13083-871, Brazil; b Laboratory of Biological Chemistry, Institute of Chemistry, UNICAMP, Campinas, SP 13083-862, Brazil; c School of Food Engineering, UNICAMP, Campinas, SP 13083-862, Brazil; d Faculty of Medicine, University of Buenos Aires (UBA), Buenos Aires C1121, Argentina; e Research Group in Scientific Computing, Information Engineering and Automation, 273992 Embrapa Digital Agriculture, Campinas, SP 13083-886, Brazil

## Abstract

Thrombosis, a critical pathological event characterized by excessive clot formation, is primarily regulated by the fibrinolytic system, where plasminogen activator inhibitors (PAIs) are pivotal. Among them, PAI-1 is the most relevant due to its strong inhibitory effect on fibrinolysis, contributing to various thrombotic disorders. In addition, PAI-2 and PAI-3 have been implicated in distinct physiological and pathological conditions. Understanding their structural and functional characteristics is essential for the development of targeted anticoagulant therapies. This study comprehensively analyzes the secondary and tertiary structures of PAI-1, PAI-2, and PAI-3, highlighting conserved domains and their roles in protein function. Comparative phylogenetic analysis confirms their evolutionary relationships, reinforcing their shared inhibitory mechanisms. Structural superimposition and root-mean-square deviation (RMSD) calculations demonstrate varying degrees of similarity among these inhibitors, with PAI-2 and PAI-3 being more closely related. Furthermore, molecular docking and molecular dynamics simulations were employed to identify natural product-derived inhibitors, focusing on tanshinones and phenolic acids extracted from *Salvia miltiorrhiza*. Among these compounds, salvianolic acid B and tanshinone IIA sulfate exhibited the highest binding affinity and stability, suggesting their potential as lead compounds for the development of novel fibrinolytic agents. Our findings contribute to understanding PAI structural dynamics and provide insights into natural inhibitor design, paving the way for innovative therapeutic strategies against thrombosis and related disorders.

## Graphical abstract

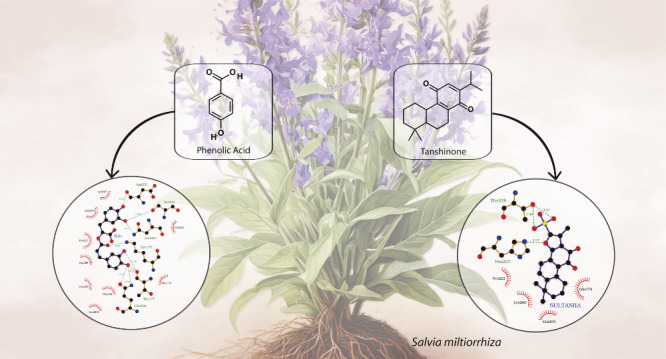

## Introduction

Blood thrombus formation, consisting of platelet aggregation and fibrinogen polymerization, is a crucial physiological process that prevents bleeding after blood vessel injury, maintaining blood in a liquid state within the vessels—a condition known as hemostasis.^1^ This process also plays a vital role in pathogen defense, a mechanism termed immunothrombosis, which benefits the host.^2^

Following vascular injury and thrombus formation, fibrinolysis initiates once the damaged endothelium is repaired, removing the fibrin plug and restoring normal blood flow. However, fibrinolysis can be disrupted in certain congenital disorders, particularly during pathological overactivation after severe injury or surgery.^3^

Intravascular fibrinolysis is primarily triggered by the incorporation of tissue plasminogen activator (t-PA) into the fibrin clot, where it converts fibrin-bound plasminogen to plasmin.^4^ While t-PA is present in normal plasma at picomolar concentrations, its activity, which determines the endogenous fibrinolysis rate, is tightly regulated by plasminogen activator inhibitor type 1 (PAI-1),^5^ a serine protease inhibitor (serpin) belonging to the serine protease family, which also includes plasminogen activators (PAs).

Though PAI-1 is the most relevant member of this family in the pathogenesis of thrombotic disorders, crucially regulating plasminogen activity and coagulation processes, other PAIs can also be found mainly in placenta (PAI-2) and urine (PAI-3). Still, lower concentration levels in blood plasma of PAI-2 and PAI-3 can be linked to other disorders such as liver cirrhosis and chronic hepatitis.^6–11^

Also, the concentration level of PAI-1 is a key factor in prothrombotic states, and its genetic regulation may be linked to 4G/5G guanosine insertion/deletion polymorphism, located at the 675th base pair upstream of the translational start point. Also, homozygosity for the 4G/4G deletion genotype results in higher PAI-1 concentration levels compared to the 4G/5G genotype.^12^

Although the clinical significance of this homozygous PAI-1 mutation alone is unclear, studies suggest an increased frequency of thrombotic events when it occurs alongside other prothrombotic conditions.^13^ Consequently, the prothrombotic state caused by the PAI-1 mutation predisposes individuals to venous and arterial thrombosis, increasing the risk of conditions like acute coronary syndrome (ACS), cerebrovascular accident (CVA), and repeated abortion (RA).^14–19^

In addition to genetic factors, epigenetic mechanisms have also been implicated in the regulation of PAI-1 and the broader hemostatic profile. Notably, the epigenetic age, estimated through DNA methylation patterns, has been associated with a prothrombotic state. Increased epigenetic age correlates with increased levels of fibrinogen and PAI-1, as well as shortened activated partial thromboplastin time (aPTT), suggesting a shift toward a hypercoagulable phenotype.^20^

Given the social effects that PAIs play in daily lives, there is no surprise in finding traditional cultures proposing the usage of natural resources that can present anticoagulant effects, such as traditional Chinese medicine that employs *Salvia miltiorrhiza* (*S. miltiorrhiza*) to treat cardiovascular and cerebrovascular diseases. The main secondary metabolites found in *S. miltiorrhiza* include two compound classes: phenolic acids and tanshinones.

Among the first class of molecules found in *S. miltiorrhiza*, one can identify salvianolic acid B (SAcB—C_36_H_30_O_16_), rosmarinic acid (RAc—C_18_H_16_O_8_), caffeic acid (CAc—C_9_H_8_O_4_), and danshensu acid (DAc—C_9_H_10_O_5_). These molecules promote fibrinolysis by activating plasminogen and inhibit fibrin formation by blocking coagulation factors, such as thrombin (factor IIa) and factor Xa.^21,22^

As for the tanshinones, there are tanshinone I (TAN—C_18_H_12_O_3_), tanshinone IIA (TANIIA—C_18_H_18_O_3_), cryptotanshinone (CRYPTO—C_19_H_20_O_3_), and dihydrotanshinone (DIHYDRO—C_18_H_14_O_3_), all of which exhibit a broad spectrum of antithrombotic activities. They can inhibit platelet aggregation by modulating receptors such as P2Y12 and reducing the level of thromboxane A2 (TXA2) signaling. These compounds also exert antioxidant effects by inhibiting pathways dependent on reactive oxygen species (ROS) and promoting the regulation of thrombomodulin, contributing to the maintenance of hemostatic balance.^23,24^

Additionally, studies indicated that a tanshinone-sulfonated analog (tanshinone IIA sulfate, SULTANIIA—C_19_H_17_O_3_SO_3_^–^) increases fibrinolytic activity by inhibiting plasminogen activator inhibitor-1 (PAI-1), favoring an environment more conducive to fibrin degradation and thrombus dissolution.^25,26^

Given the involvement of PAIs in various pathological processes, research into their structure and inhibition is crucial for developing potential drugs, such as that of Li et al.^27^ Authors^27^ studied the interaction between the inhibitors CDE-096 and PAI-1, using X-ray crystallography and mutagenesis to characterize an allosteric mechanism of inhibition based on the compound’s binding to the sB/sC site of the protein. These structural approaches revealed that CDE-096 prevents PAI-1 from binding to both proteases and the cofactor vitronectin, demonstrating the potential of serpin inactivators with high specificity.

In addition, the study by Izuhara et al.^28^ employed virtual screening to identify new PAI-1 inhibitors, highlighting the usefulness of *in silico* methods in discovering molecules capable of inhibiting serpin activity and potentially enhancing fibrinolysis. Together, these studies exemplify how *in silico* strategies can be integrated into rational drug design, providing insights into the structure and function of target proteins and facilitating the development of new inhibitors.

Herein, this work compares the secondary and tertiary structures of PAI-1, PAI-2, and PAI-3, analyzing their domains, functions, and structural conservation. We also explored putative mutations and assessed potential inhibitor drugs from natural compounds found in *S. miltiorrhiza*, showing the phenolic compound RAc as a potential candidate for the specific inhibition of PAI-1.

## Results and Discussion

### Phylogenetic Processing Step

Over our search on digital protein platforms, we have found that PAI-1 (UniProt ID: P05121), PAI-2 (UniProt ID: P05120), and PAI-3 (UniProt ID: P05154) have other names such as SERPINA 1, SERPINA B2, SERPIN A5, or Protein C Inhibitor (PCI), respectively. Unlike PAI-1, which is secreted by endothelial cells, and can act as an inhibitor of tissue plasminogen activators (t-PA) and urokinase (u-PA),^29^ PAI-2 is secreted by monocytes and can inhibit just the u-PA. Therefore, the two enzymes are synthesized within their respective cells, but are excreted into the bloodstream,^30^ and show different activities. While PAI-3 may play a role similar to that of PAI-1, it also inhibits blood clotting factors such as prothrombin, factor XI, factor Xa, plasma kallikrein, and fibrinolytic enzymes. Nevertheless, PAI-3 can act as a procoagulant and proinflammatory factor, inhibiting activated protein C anticoagulant factor and the generation of activated protein C factor by the thrombin/thrombomodulin complex. PAI-3 is the only enzyme among human PAIs dependent on another factor, heparin. PAI-3 can be found in biological fluids such as urine, saliva, amniotic fluid, seminal plasma, and human seminal vesicle secretions, the last two showing higher concentrations of PAI-3.^31^

Concerning the enzyme structure, after the PAI-1 mRNA is translated into the mature protein, it will show 402 aa residues, with 23 residues forming the signal peptide and the remaining 379 residues forming the active protein, containing three glycosylation sites (N209, N265, and N329).^8^ PAI-3 also presents modifications, being formed by 406 aa residues, with 19 residues forming the signal peptide, and the remaining 384 residues forming the active protein, which also have at least three glycosylation sites (N230, N243, and N319).^32,33^ Unlike PAI-1 and PAI-3, after translation, PAI-2 presents an internal signal peptide, totaling 415 aa residues. However, like PAI-1 and PAI-3, PAI-2 has three glycosylation sites (N075, N115, and N339).^34^

As stated before, the three PAIs are from the serpin family, and PFam searches confirm that PAI-1 belongs to such a family with an E-value of 7.8*e* ^–108^. Interestingly, PAI-2 PFam shows that it could belong to two families: serpin and GDE_C (amyl-alpha-1,6-glucosidase - human glycogen branching enzyme), with *E*-values of 8.4*e* ^–130^ and 0.24, respectively. As the former is much lower than the latter *E*-value, we can say that PAI-2 belongs only to the serpin family. A similar occurrence was seen for PAI-3, which could belong to serpin, YfdX (YfdX protein—is a protein found in *Proteobacteria* of unknown function), or NOPS (NOPS—found in the C-terminus of NONA and PSP1 proteins) families with *E*-values of 3.2*e* ^–116^, 0.2, and 0.43, respectively. Therefore, we can also say that PAI-3 belongs to the serpin family.

Members of the serpin family exhibit extensive flexibility and polymorphism, particularly in their active site segments and in the β-sheet secondary structure, which can absorb and expel strands.^35,36^ Once aligning the aa residues of all PAIs taken from UniProt, by employing the Clustal online platform Omega, we have found 48 conserved residues indicated by (*), 85 residues with strong similar physicochemical properties being indicated by (**:**), and 37 residues with weak similar physicochemical properties being indicated by (.), as illustrated in Figure 1a.

**Figure 1:**
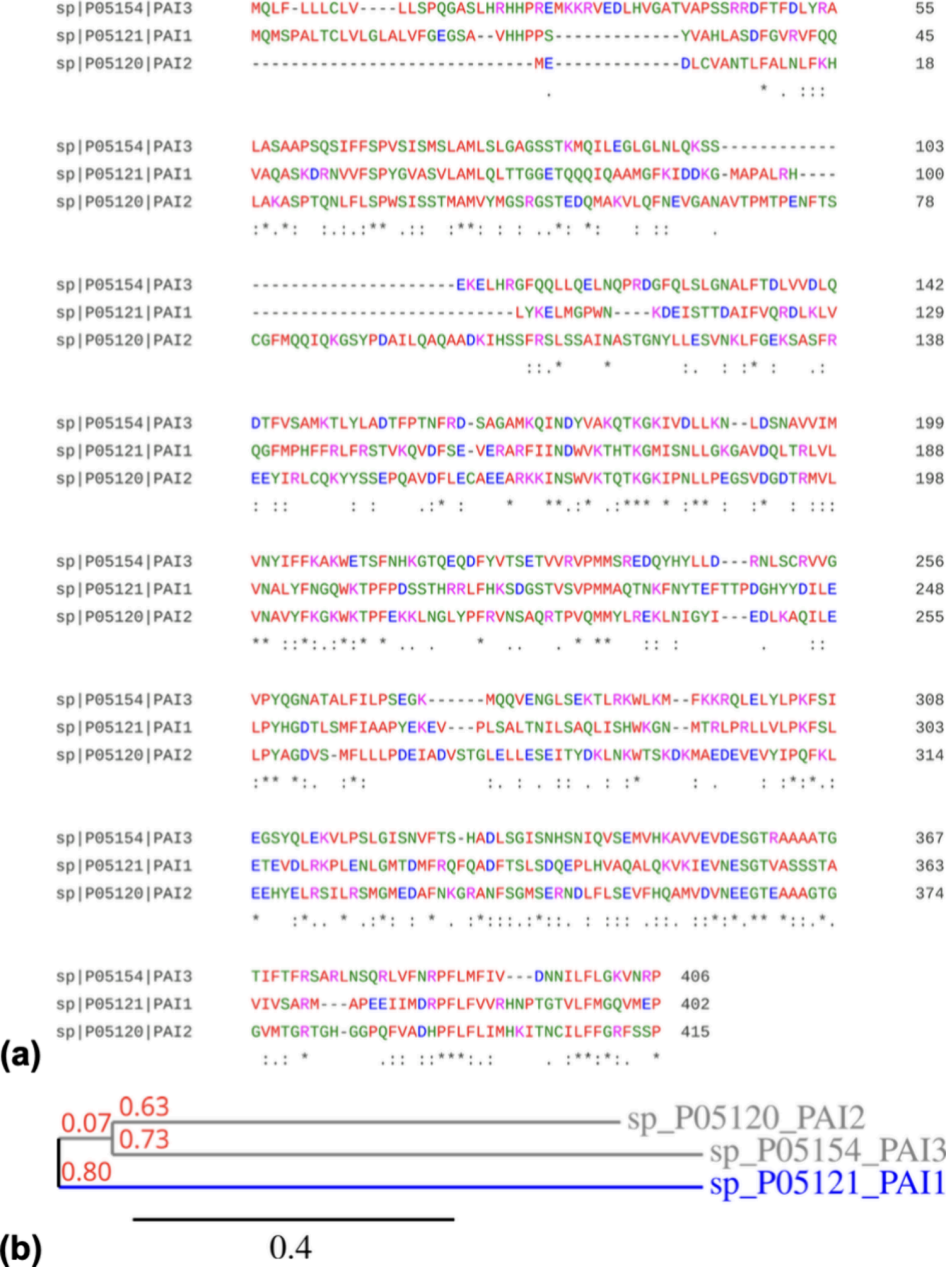
(a) Alignment of the PAI aa sequences, with small residues in red, acidic residues in blue, basic residues in magenta, and other residues in green. (b) Phylogeny of the PAIs obtained from the phylogeny.fr online platform.

On one hand, while proteins related by homology, i.e., with similar evolutionary origin, are called homologous,^37^ the orthologs are related via speciation, *i.e*., proteins that had their function kept after an evolutionary event that led to the dawn of two different species. On the other hand, paralog proteins are related via duplication, which can happen in an organism in a way that protein function is not kept identical.

The combination of speciation and duplication events, along with horizontal gene transfer (HGT), gene loss, and gene rearrangements, entangle orthologs and paralogs in complex webs of relationships.^37^ Knowing this, and analyzing data (Figure 1b), it can be concluded that the three PAIs are homologous proteins, but all of them are orthologous, as they perform the same role, suggesting that the PAIs may not be considered paralogs.

### Structural Analysis

From the structural perspective, according to Wright,^35^ proteins belonging to the serpin family have their active site between two β-sheets. These β-sheets have inherent plasticity, which explains the polymorphism of the active site in serpins. Nevertheless, that would explain their inhibitory properties, in addition to biological aggregation. To assess the structural characteristics of the PAIs, such as the motifs and the secondary structures of the PAIs, we selected on the PDBSum platform the highest crystallographic resolution, which led us to a PAI-1 structure with a resolution of 1.77 Å (PDB ID: 7AQF; 379 aa), PAI-2 (PDB ID: 2ARR; 382 aa), and PAI-3 (PDB ID: 3DY0; 336 aa) structures, the last two presenting a resolution of 1.55 Å. Hence, Table 1 summarizes the PAIs’ secondary structures along with the found motifs.

**Table 1:** Quantities of Secondary Structures in PAI-1 (PDB ID: 7AQF), PAI-2 (PDB ID: 2ARR), and PAI-3 (PDB ID: 3DY0) Enzymes

Structures	PAI-1	PAI-2	PAI-3
β-sheets	16	19	14
α-helices	11	11	12
motifs	PAI-1	PAI-2	PAI-3
β hair clips	5	5	5
β bumps	4	5	3
α-α	12	12	13
β turns	32	31	22
turns γ	2	4	1
turns ψ	1	1	1
β–α–β (active site location)	1	1	1

Nevertheless, we also compared the 3D structure similarity of the PAIs by computing the RMSD of their superimposition, which was taken by considering an alignment between all atoms, only the α carbons (C_α_–C_α_), and only the carbon atoms of all aa (C–C). Figure 2 depicts the superimposition results of the PAI structures, and Table 2 shows the RMSD values of all alignment scenarios.

**Figure 2:**
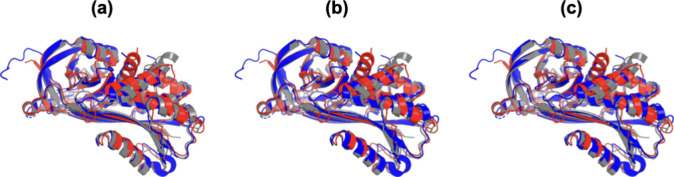
Superimposition of PAI structures 1, 2, and 3, in red, blue, and gray, respectively; motif β–α–β at the active site can be seen in the images. Superimposition representations are for (a) all superimposable aa atoms, (b) only the C_α_ atoms of the backbone, and (c) only the C atoms of the aa residues.

**Table 2:** RMSD Values of the Three Superimposed Structures for Each Superimposition Method

Reference	Target	RMSD (Å) [all atoms]	RMSD (Å) [C_α_–C_α_]	RMSD (Å)[C–C]
PAI-1 (7AQF)	PAI-2 (2ARR)	2.033	1.411	1.292
PAI-1 (7AQF)	PAI-3 (3DY0)	1.866	1.441	1.472
PAI-2 (2ARR)	PAI-3 (3DY0)	0.913	0.850	0.859

According to Damm and Carlson,^38^ a good RMSD match for structural similarity ranges from 0 to 5 Å. Therefore, the RMSD results (Table 2) suggest that PAI-2 and PAI-3 are more closely related than PAI-1 is to PAI-2 or PAI-3, which is also confirmed by the phylogeny data (Figure 1b). In addition, one interesting feature of the PAI structures that is not easily seen in Figure 2 is a loop motif known as the reactive center loop (RCL),^39^ which is found next to the binding pocket of the PAIs.

The missing RCL for PAI-1 and PAI-2 is found from V_334_ to P_349_ and from G_366_ to T_378_, respectively. As for PAI-3, this motif is not verified, agreeing with the peculiar difference between PAI-3 and the two other PAIs, as highlighted by the phylogeny analysis. Also, as the loop is known for its high flexibility, with an important role in making the PAI activity functional, it can be difficult to use the usual crystallographic techniques to capture its structure.

As PAI-1 is a serpin that undergoes conformational changes between active, latent, and substrate forms, one can easily understand how crucial these transitions are for its function as an inhibitor of tissue-type plasminogen activator (t-PA) and urokinase-type plasminogen activator (u-PA). For instance, the active form of PAI-1 forms a stable complex with its target proteases, while the latent form does not, and the substrate form is cleaved by the protease.^40,41^ The structural basis for these transitions involves the insertion of the RCL into beta-sheet A, which is a common feature among serpins.^41,42^

PAI-2 also belongs to the serpin family and shares the same mechanism of insertion of RCL into beta-sheet A upon interaction with proteases. However, PAI-2 has distinct structural features that influence its stability and function. The crystal structure of PAI-2 reveals a disordered RCL in its stressed state, and a polar cluster beneath beta-sheet A stabilizes both the stressed and relaxed forms through hydrogen bond rearrangements.^30^ This structural arrangement is critical for its inhibitory function and distinguishes it from PAI-1.

However, PAI-3, also known as a protein C inhibitor, is less studied in the context of these conformational changes promoted by RCL, but it is known to have unique structural motifs that differentiate its function from PAI-1 and PAI-2. The specific secondary structures and motifs of PAI-3 contribute to its role in regulating protein C and other serine proteases.

### Mutations—Studies

Additionally to the RMSD and phylogeny agreement, both UniProt and PDBSum pointed out that PAI-1 (7AQF) can display common mutations at the following residues: T_184_→I_184_, R_186_→H_186_, N_209_→S_209_, and T_232_→N_232_. Those mutations play an important role not only in distinguishing PAI-1 from the other PAIs but also in stabilizing the PAI-1 structure and leading the structure to a more rigid system, as the DynaMut data showed (Table 3).

**Table 3:** Free Energy (ΔΔ*G*) and Vibrational Entropy (ΔΔ*S*) Variation Values of the Mutations Predicted by the DynaMut Server^a^

parameter	T_184_→I_184_	R_186_→H_186_	N_209_→S_209_	T_232_→N_232_
ΔΔ*G*_DynaMut_	1.642	1.216	2.069	1.397
ΔΔ*G*_ENCoM_	2.447	2.356	2.515	2.391
ΔΔ*G*_SDM_	1.120	0.270	–0.490	0.460
ΔΔ*G*_mCSM_	–0.019	–0.730	–1.439	–0.789
ΔΔ*G*_DUET_	0.502	–0.498	–1.253	–0.439
ΔΔS_vibe‑ENCOM_	–3.059	–2.944	–3.144	–2.989

a Values are given in kcal mol^–1^ and kcal mol^–1^ K^–1^, respectively.

On one hand, according to thermodynamics, positive ΔΔ*G* values indicate that the mutation is stable, and negative ΔΔ*G* values mean that the mutation is unstable. Roughly speaking, the DynaMut data points out that all mutations are stabilizing, which is in agreement with other methods (ENCoM, SDM, and DUET), except for CSM. On the other hand, the variation in vibrational entropy term (ΔΔS_vibe_) describes how flexible the structure becomes; that is, negative values of ΔΔ*S*_vibe_ indicate higher rigidity, as positive values indicate higher flexibility. Hence, the data shown in Table 3 point to a flexibility loss. The stability and rigidity increases are related to the change in the interaction contact net promoted by the mutations leading to a local rearrangement of interactions over the structure; that is, the modification of the number of noncovalent interactions going from the wild type to the mutant one is noticeable, as shown in Figure 3.

**Figure 3:**
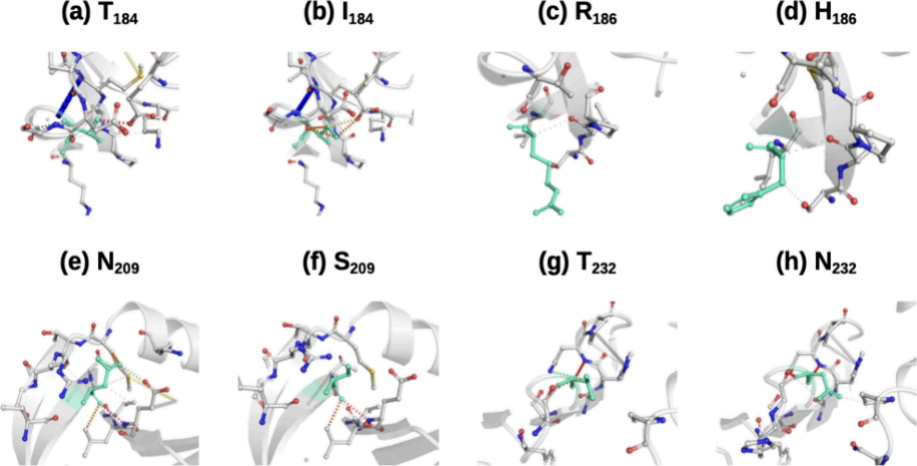
Wild and mutants of aa according to DynaMut for 7AQF, ranging from (a) to (h): T184→I184, R186→H186, N209→S209, and T232→N232.

Despite leading to more stable structures, these mutations yield an increase of the PAI-1 rigidity, which results in a nonfunctional structure, as the RCL is the most affected by these mutations, as shown by the red portion of the blue cartoon tube in Figure 4.

**Figure 4:**
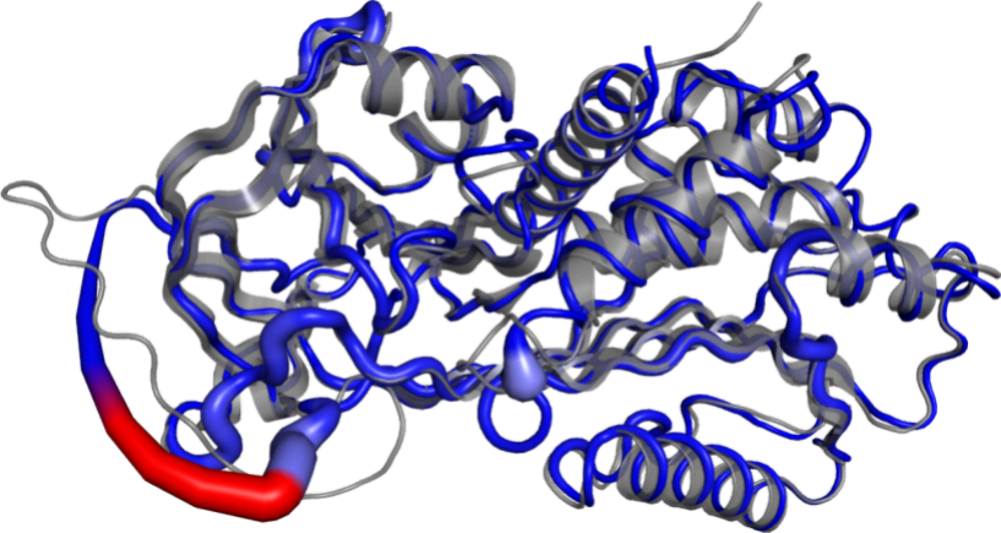
Rigidity heat map of the PAI-1 structure. Cartoon blue tube displaying the mutant type with the red (stronger) and light blue (rough) indicating the degree of rigidity increase. The cartoon structure in gray color depicts the wild type.

In addition to these stability-related mutations, a clinically relevant variant involving a highly conserved glycine residue in strand 5B (Gly397) has been reported. This glycine plays a crucial role in maintaining the structural packing of β-sheet B in PAI-1 and is conserved across the SERPIN family due to its minimal steric hindrance. A substitution of Gly397 with a bulkier, positively charged arginine (G397R) disrupts this tightly packed region, leading to abnormal polymerization of the protein within the cell. As a result, mutated PAI-1 fails to be secreted and accumulates intracellularly, causing a severe functional deficiency. This polymerization mechanism is similar to other serpinopathies, such as those involving antithrombin III and neuroserpin, and has been linked to life-threatening bleeding episodes due to uncontrolled fibrinolysis in affected individuals.^43^

Another structural determinant of PAI-1 stability was uncovered in a mutant bearing four substitutions: Asn150→His, Lys154→Thr, Gln319→Leu, and Met354→Ile. Although these mutations appeared to act cooperatively, only the K154T and Q319L mutations significantly contributed to the increased stability. In particular, the K154T mutation induced a conformational rearrangement in the loop connecting α-helix F and β-strand 3A (hF-s3A loop). This shift enables the formation of several new hydrogen bonds between the loop residues and elements of β-sheet A and helix F. These new interactions create a more interconnected and rigid core, which likely explains the enhanced structural stability of the mutant compared to the wild-type protein.^44^

Furthermore, mutagenesis of other residues in surface-exposed regions of PAI-1, such as R101, M110, and Q123 (R101A-M110A-Q123A mutant), resulted in a complete loss of vitronectin binding. Interestingly, this triple mutation did not compromise the protease inhibitory function or receptor interactions of PAI-1, but it demonstrates how discrete residue changes can selectively affect binding partners and functional specificity.^44^

### Docking and Molecular Dynamics

Since the chosen models of PAI-1 (7AQF), PAI-2 (2ARR), and PAI-3 (3DY0) are apo structures, we took a holo PAI-1 model (4G8O) and aligned it with the chosen apo PAIs’ models. Through that strategy, we found that the binding pocket of PAI-1 (7AQF) and PAI-2 (2ARR) comprises a total of 11 aa, as PAI-3 (3DY0) totalizes 13 aa. Yet, the binding pocket of all of the PAI structures seems to structurally conserve at least seven aa, Glu, Leu, Lys, Phe, Pro, Thr, and Tyr, as shown in Figure 5.

**Figure 5:**
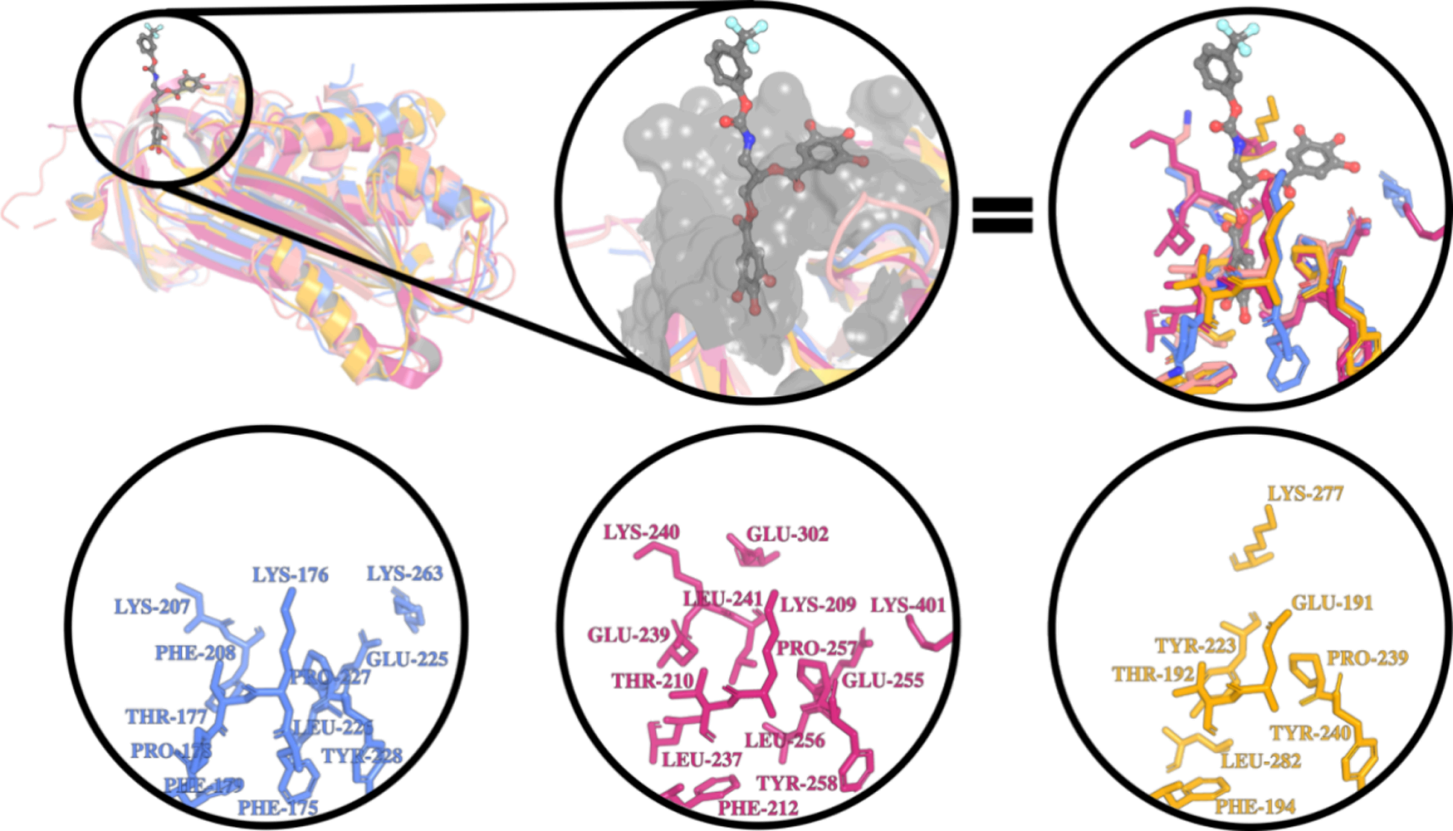
Binding pocket selection through Cα backbone superposition of 4G80 (pink), 7AQF (blue), 2ARR (magenta), and 3DY0 (yellow). Highlighted in dark circles at the top: the 4G80 ligand, zooming at the surface rendering of the pocket’s surface in gray color, and sticks depicting the conserved aa from each PAI. The bottom circles depict the aa residues isolated from the pocket for the 7AQF (blue), 2ARR (magenta), and 3DY0 (yellow), respectively.

As the crystallographic structures were missing some aa for the RCL, we submitted the structures to AlphaFold for generating new models with the current missing aa in place, which returned five different models for each PAI structure. Although the AlphaFold models showed a minimal RMSD deviation, the model selection based on the lowest RMSD as shown in Table 4 led us to the second model with the minimum error concerning the original structure for PAI-1 and PAI-3; as for PAI-2, the first model showed that pattern.

**Table 4:** RMSD Values for the Alignment between the AlphaFold Models and the Original Structures, Lowest RMSD Values Marked with *

	RMSD (Å)				
PAI	model 1	model 2	model 3	model 4	model 5
PAI-1 (7AQF)	0.586	0.562*	0.606	0.587	0.573
PAI-2 (2ARR)	0.530*	0.584	0.635	0.617	0.531
PAI-3 (3DY0)	0.396	0.392*	0.406	0.464	0.447

Once the models were determined, we prepared the ligands and the positive control (tiplaxtinin)^28^ for the docking campaign, leading to the results summarized in Table 5. According to our docking results, RAc stands out as the lowest value for PAI-1, meaning that its binding strength to the PAI-1 pocket is the highest of all scenarios except that for the positive control.

**Table 5:** Docking Affinity Energy (*E*_aff_), Average Hydrogen Bond Distances (Avg_HBd_), Average Hydrogen Bond Angle (Avg_HBa_), and Averaged Surface Contact Area of Hydrophobic Interactions (*S*_area_)­^a^

	*E*_aff_ (kcal mol^–1^)			Avg_HBd_ (Å)			Avg_HBa_ (°)			*S*_area_ (Å^2^)		
ligands	PAI-1	PAI-2	PAI-3	PAI-1	PAI-2	PAI-3	PAI-1	PAI-2	PAI-3	PAI-1	PAI-2	PAI-3
CAc	–6.70	–5.00	–4.40	2.04 (3)	2.32 (4)	2.18 (3)	156	137	143	234.21 (9)	225.73 (6)	218.44 (3)
DAc	–6.20	–5.10	–4.50	2.47 (1)	2.61 (4)	2.13 (4)	125	152	147	251.07 (5)	235.92 (6)	219.73 (6)
RAc	–8.10	–6.60	–5.50	2.26 (6)	2.50 (3)	2.22 (4)	129	122	129	376.35 (9)	379.78 (9)	334.20 (3)
SAcB	–7.40	–6.20	–5.70	2.25 (7)	2.48 (5)	2.22 (10)	144	139	138	512.27 (8)	439.07 (9)	383.56 (3)
CRYPTO	–5.30	–5.90	–5.00	2.19 (2)	2.28 (1)	- (−)	127	123		282.01 (5)	273.29 (10)	242.54 (6)
DIHYDRO	–6.10	–5.60	–5.00	2.13 (1)	2.34 (2)	2.37 (1)	135	130	99	273.11 (8)	290.04 (7)	231.49 (6)
TAN	–6.10	–5.40	–4.80	2.02 (1)	- (−)	- (−)	138			262.47 (8)	253.19 (10)	250.63 (6)
TANIIA	–5.70	–5.20	–5.00		- (−)	- (−)				267.05 (8)	270.13 (9)	246.63 (5)
SULTANIIA	–6.10	–5.40	–6.50		2.51 (2)	2.19 (2)		155	132	296.78 (4)	328.46 (8)	277.79 (4)
tiplaxtinin	–8.20	–6.30	–5.50	1.92 (1)	2.11 (1)	- (−)	157	156		401.86 (9)	407.81 (10)	335.79 (8)

a Number of hydrogen bonds and polar contact counts shown in parentheses after Avg_HBd_, and *S*_area_, respectively. Tiplaxtinin is shown as positive control.

On the other hand, for PAI-2, RAc stands as a good candidate for a strong binding mechanism, stronger than that seen for the control; as for PAI-3, SULTANIIA seems to present the best result instead of RAc, suggesting that RAc may not be as good as SULTANIIA for inhibiting PAI-3. Overall, RAc might not work as a silver bullet for all PAIs, but it seems to have promising inhibition potential over PAI-1 and PAI-2.

Nevertheless, the docking results point to a complex web of interactions between the ligands and the binding pockets of the proteins; each target molecule showed a varying value of hydrogen bonds (HB) contacts, distances (HBd) and angles (HBa), hydrophobic contacts (HC), and contact surface area for the HC interactions (*S*_area_). It might be tempting to directly suggest that the higher the number of HB, PC, and S_area_, the lower the binding affinity (*E*_aff_) of the ligand to the protein’s pocket. However, it is clearly observable that SAcB (Table 5) should present the best result for all scenarios, which is not confirmed by the *E*_aff_ value.

This SAcB behavior may be explained by an indirect measure of the noncovalent interaction strength, *i.e.*, hydrogen bonds and polar interactions. According to Kraka et al.,^45^ an alignment of the HBa closer to 180° and HBd closer to 2.5 Å would lead to a stronger interaction between the ligand and the protein’s pocket. Hence, SAcB would be a good match according to HBd and HBa, with values slightly better than other ligands, thus indicative for the best ligand candidate.

However, as demonstrated by Kurgan and Chen,^46^ the majority of the governing forces between proteins and organic molecules are of van der Waals nature, meaning that this kind of interaction should be taken into account once assessing the docking *E*_aff_. Hence, by considering the population density of HC in a given *S*_area_, it is possible to observe a slight change in the previously seen pattern, and SAcB is not always the best candidate.

In order to illustrate these concepts, we depict in Figure 6 the highest and lowest *E*_aff_ energies for each PAI structure. One can easily see that for PAI-1, the highest affinity energy, given by ligand CRYPTO, shows a total of 5 HC interactions distributed in an *S*_area_ of about 282 Å^2^ and 2 HB; as for the lowest-affinity energy given by ligand RAc, one can count 9 HC within 376 Å^2^ and 6 HB.

**Figure 6:**
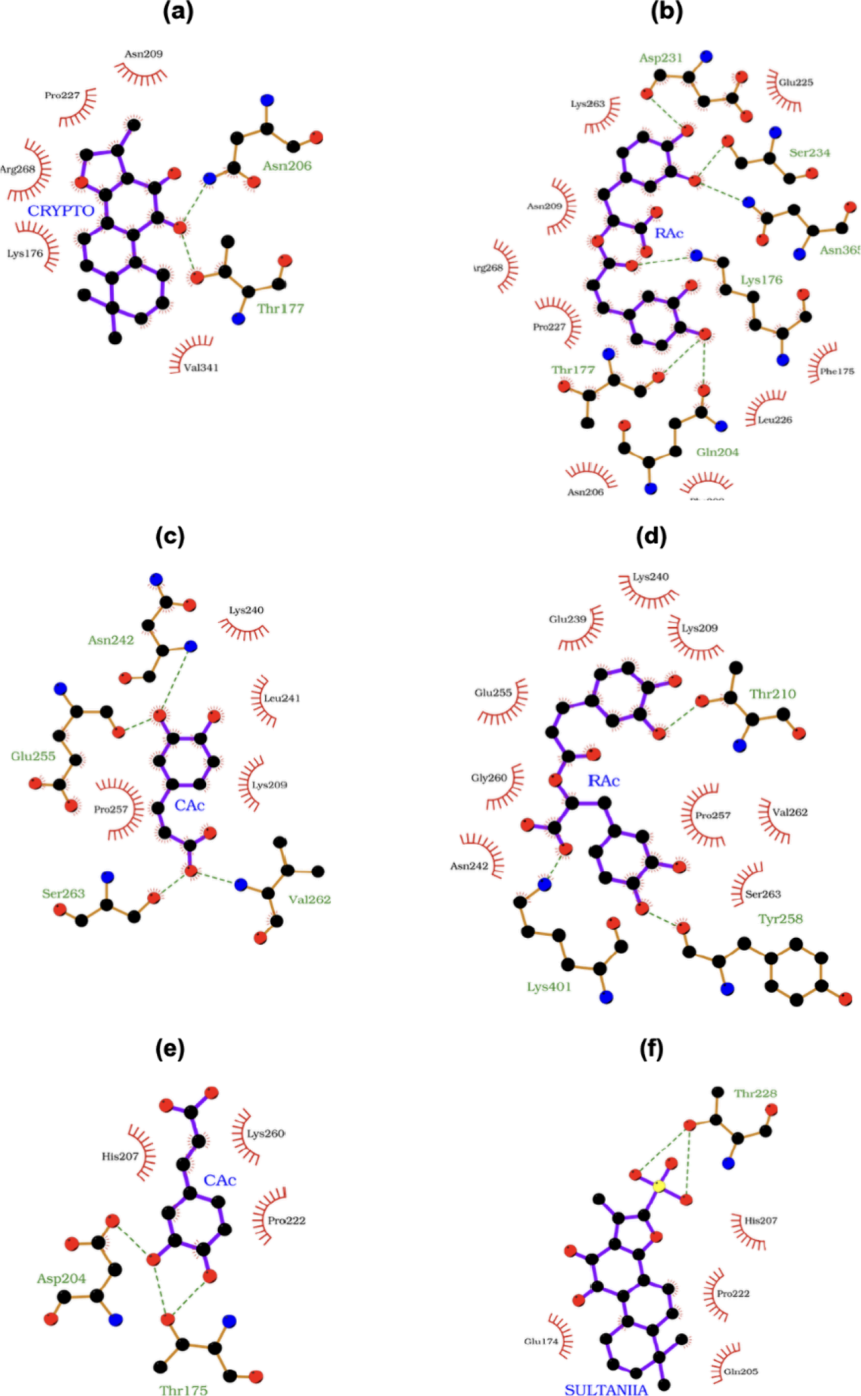
Docking poses for (a) PAI-1 and CRYPTO, (b) PAI-1 and RAc, (c) PAI-2 and CAc, (d) PAI-2 and RAc, (e) PAI-3 and CAc, and (f) PAI-3 and SULTANIIA. Red semicircles depict HC interactions, as dashed green lines indicate hydrogen bondings. Carbon, oxygen, nitrogen, and sulfur are colored black, red, blue, and yellow, respectively. Ligand bonds are depicted in violet, and aa bonds are in pale yellow. Hydrogens are hidden to provide a cleaner view.

Interestingly, a close inspection of the results depicted in Figure 6 may lead to an odd rationale for explaining why SULTANIIA (Figure 6f) has an affinity energy lower than that of CAc (Figure 6e), despite a higher number of hydrogen bonds observed for CAc. The reason for that is simply because the ligand structure of SULTANIIA has stronger hydrogen bonds as it has an ionized group (−SO_3_^–^) that promotes a greater polarization. Also, the ligand has a greater *S*_area_ than that of CAc, allowing it to better distribute more HC interactions over its surface, making it easier for the ligand to sit in the binding pocket, and even leading to slightly denser HC interactions. That is, though the number of HB interactions for SULTANIIA is lower than that seen for CAc, SULTANIA interactions are stronger and occupy a greater spatial range.

Hence, the docking results suggest that RAc and SULTANIIA might be excellent candidates for acting as inhibitors of PAIs. That was assessed through the molecular dynamics simulation, which allowed us to analyze the RMSD of the protein and ligand structures over the course of the simulation time, concerning its initial pose, as shown in Table 6. Clearly, SULTANIIA, when bound to PAI-3, showed the lowest RMSD value averaged, followed by RAc once bound to PAI-2 and then PAI-1. These results might suggest that the proposed ligands may have the desired behavior as inhibitors but, by analyzing the RMSD distribution, as shown in Figure 7, may suggest a slightly different answer.

**Table 6:** Average and Standard Deviation (Std.) of the RMSD Values Given in Å for the Protein Structures and the Ligands over the Course of the Simulation Time

structure	average	std.
PAI-1	0.25	0.04
PAI-2	0.48	0.08
PAI-3	0.54	0.06
PAI-1-RAc	0.22	0.04
PAI-2-RAc	0.18	0.04
PAI-3-SULTANIIA	0.07	0.02

**Figure 7:**
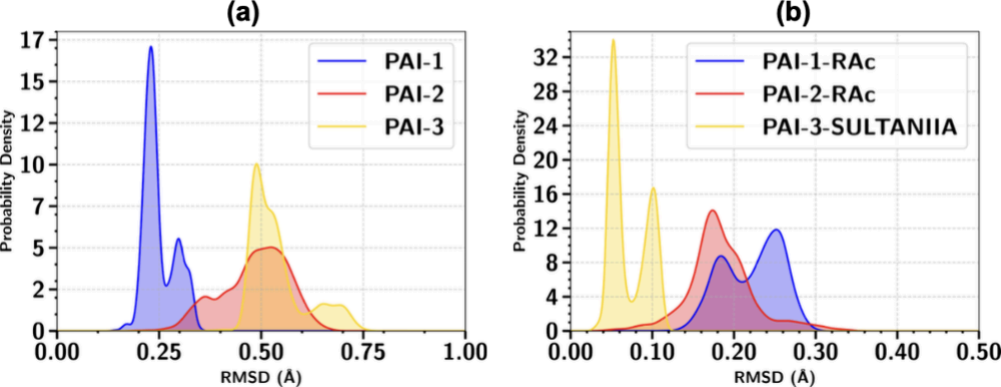
Distribution of the RMSD for the PAI structures (a) and the ligand structures (b).

Investigating Figure 7a features, there is a broad band for PAI-2 and two slightly well-defined bands for PAI-3, suggesting that these structures may suffer a more significant structure instability than that seen for PAI-1, with two sharp band peaks being well-defined. As for the ligands depicted in Figure 7b, SULTANIIA seems to be a more stable ligand, suggesting that it stays for longer periods in almost two preferential forms, while RAc in PAI-2 seems to delocalize itself over the course of the simulation time; as for PAI-1, it may have two distinguishable forms, suggesting a slightly stable inhibitor than that seen in PAI-2.

On the other hand, analyzing carefully data illustrated in Figure 8a, it is clear that the main reason for the two peaks seen in Figure 7a for PAI-1 might be related to the opening of the RCL as it is kept relatively closed over the course of the first 40 ns, showing signals of opening after the 60 ns of the simulation time. Yet, in Figure 8b, for PAI-2, it is clear that over the course of all simulation times, the protein structure and the ligand positioning are not stable, showing great changes that may be overlooked by a simple average measure. Finally, Figure 8c depicts well how stable the complex protein–ligand system is with an almost negligible RMSD variation over time, as highlighted by the moving average in the graph.

**Figure 8:**
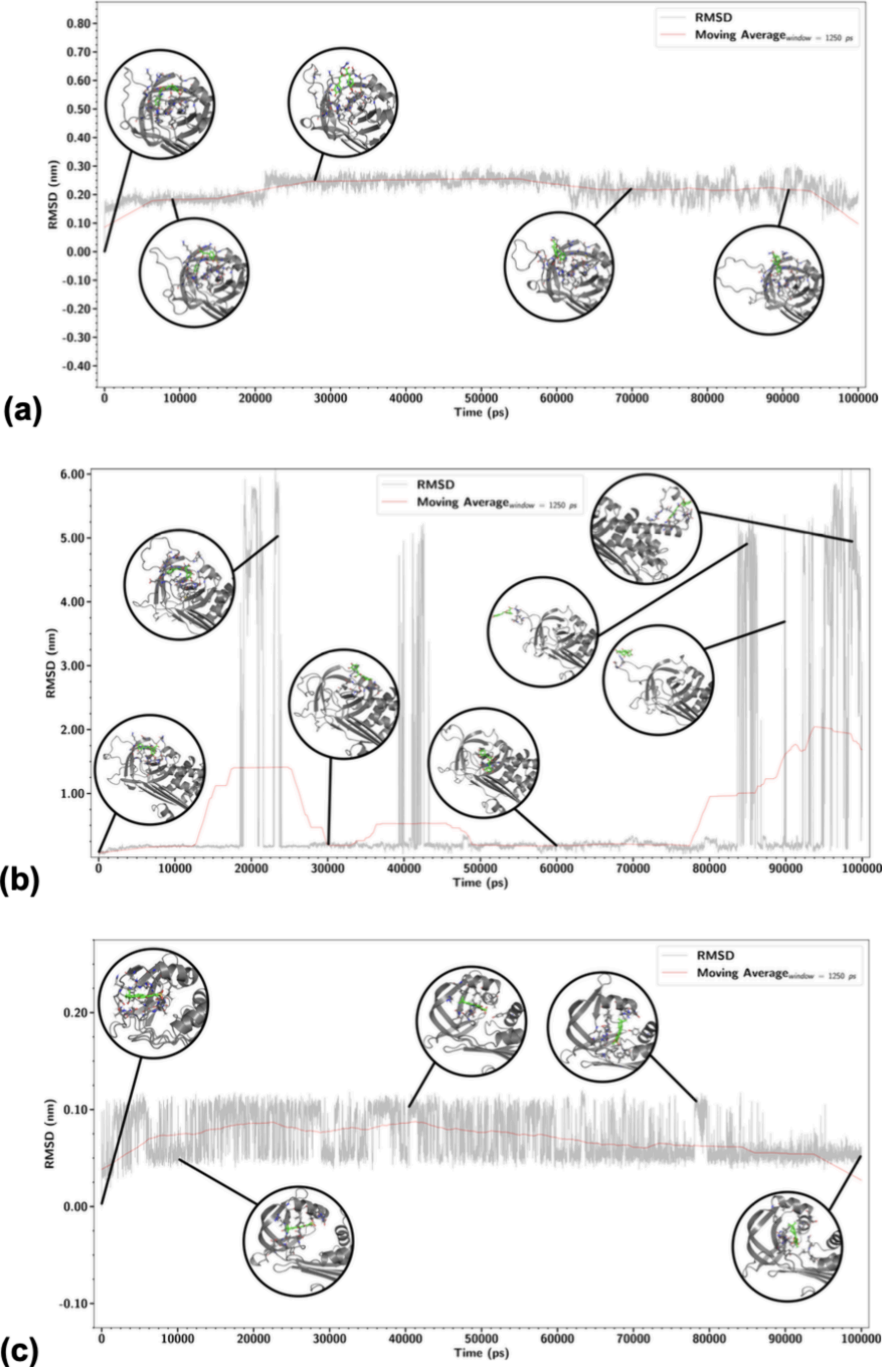
Measurement of the ligand RMSD over the molecular dynamics simulation course within 100 ns, moving average set to a window of 1250 ps shown as a red line. Simulation of PAI-1 with RAc (a), PAI-2 with RAc (b), and PAI-3 with SULTANIIA (c).

In addition to the RMSD analysis, the decomposition of the free energy for the total simulation time of each complex protein–ligand system revealed interesting features. Overall, as shown in Table 7, it seems clear that PAI-1 and PAI-3 present similar binding free energy values (Δ*G*), once considered the standard deviations, as PAI-2 showed a higher value with a great error associated with it, meaning that PAI-2 and RAc indeed form the weakest complex formed from the three scenarios, in agreement with the previously suggested RMSD analysis.

**Table 7:** Averaged (Avg.) and Standard Deviations (Std.) of the Free Energy Decomposition for the Complex Protein–Ligand System for All PAIs^a^

	PAI-1-ACr		PAI-3-ACr		PAI-3-SUL	
energy component	avg.	std.	avg.	std.	avg.	std.
Δ*E*_vdW_	–19.97	0.94	–12.15	1.53	–28.92	1.06
Δ*E*_EL_	–127.59	0.21	22.40	5.21	–7.07	0.65
Δ*E*_GB_	127.01	2.34	–19.45	2.81	18.95	0.69
Δ*E*_Surf_	–4.13	0.24	–2.53	0.37	–3.84	0.03
Δ*G*_gas_	–147.55	1.44	10.24	5.52	–35.99	1.60
Δ*G*_solv_	122.88	2.35	–21.98	2.84	15.10	0.69
Δ*G*	–24.68	2.75	–11.73	6.21	–20.89	1.74

a Values are given in kcal·mol^‑1^. SULTANIIA is abbreviated to SUL.

However, a close inspection of the energy components of each complex system points to a very unique set of driving forces for PAI-1-RAc and PAI-3-SULTANIIA. While the former shows strong negative values for electrostatic contribution (Δ*E*_EL_) and a mild contribution of van der Waals energy (Δ*E*_vdW_), the latter depicts almost an opposite contribution net. These findings suggest that hydrogen bonds may be the driving force behind PAI-1-RAc interactions, while in PAI-3-SULTANIIA, the hydrogen bonds might not be as relevant as that for PAI-1-RAc, though it was expected that the putative hydrogen bonds seen in SULTANIIA would be stronger due to the polarization expected by the ionized SO_3_^–^ group.

Nevertheless, despite a stronger electrostatic contribution seen in PAI-1-RAc, it is noticeable that the binding of RAc in PAI-1 is very unfavorable than that seen for PAI-2-RAc or PAI-3-SULTANIIA, according to the generalized born electrostatic solvation energy (Δ*E*_GB_). Yet, even though PAI-2-RAc showed a negative value for Δ*E*_GB_, RAc seems to be repealed by the binding pocket of PAI-2 according to Δ*E*_EL_. That repealing behavior is in agreement with the observed broader band of the ligand distribution RMSD (Figure 6b), as well as the many RMSD peaks seen in Figure 7b. In other words, RAc does not bind effectively to PAI-2, and despite its negative Δ*G*, it only presents a mild affinity to the overall surface of PAI-2, while for PAI-1, it signals more specificity and stability toward the maintenance of the ligand within the binding pocket, as does SULTANIIA to PAI-3.

## Conclusions

Plasminogen activator inhibitors (PAIs) are pivotal regulators of the fibrinolytic cascade, with PAI-1 being the principal physiological inhibitor of tissue-type and urokinase-type plasminogen activators. In this study, a comprehensive structural and functional analysis of PAI-1, PAI-2, and PAI-3 was conducted, revealing conserved serpin features and domain architecture while also highlighting differential evolutionary and structural attributes. Phylogenetic and RMSD-based structural superposition analyses demonstrated that PAI-2 and PAI-3 are more closely related to each other than to PAI-1, consistent with their functional divergence.

Through *in silico* mutagenesis, we identified key residues in PAI-1 whose substitutions enhance structural rigidity and thermodynamic stability but compromise conformational flexibility—particularly within the reactive center loop (RCL), a motif essential for protease inhibition. These mutations, despite being stabilizing, may hinder the transition between active and latent forms, thereby impairing the biological activity.

To explore the therapeutic modulation of the PAI function, we employed molecular docking and molecular dynamics simulations using natural products derived from *S. miltiorrhiza* including phenolic acids and tanshinones. Among the tested ligands, rosmarinic acid (RAc) exhibited the highest binding affinity and stability toward PAI-1, primarily driven by electrostatic interactions and hydrogen bonding. Conversely, the sulfonated derivative tanshinone IIA sulfate (SULTANIIA) demonstrated superior interaction with PAI-3, mediated by favorable van der Waals forces and enhanced polarizability due to the ionized sulfate group.

Binding free energy decomposition via MM/GBSA analysis supported these findings, indicating distinct interaction profiles: PAI-1–RAc complexes were dominated by electrostatics, whereas PAI-3–SULTANIIA complexes were stabilized primarily through dispersion forces. Although RAc also showed a moderate affinity for PAI-2, increased conformational fluctuation of the ligand and protein throughout the simulation suggested a less favorable interaction.

In summary, this integrative study provides structural and mechanistic insights into PAI regulation and inhibition, highlighting RAc and SULTANIIA as promising scaffold candidates for the development of isoform-selective fibrinolytic agents. These findings lay a theoretical foundation for subsequent *in vitro* and *in vivo* validation and underscore the potential of natural product-inspired anticoagulant therapies.

## Material and Methods

### Phylogenetic Processing Step

The search for PAI-1, -2, and -3 was carried out on the UniProt online platform, from which it was obtained information on function, name, taxonomy, cellular localization, phenotypes, variants, post-translational changes, expression, interactions, 3D structure, amino acid (aa) sequence, and similar proteins [https://www.uClustalniprot.org/].

Data about the family and domain were searched on the PFam online server (https://pfam.xfam.org/search/sequence), in which the expectation value (*E*-value) was employed to decide, for the studied proteins, the significance of family matches, i.e., how close a family group is to a given protein. The lower the *E* values, the higher the match to a certain family.

The aa sequences of all PAIs were subjected to alignment on the Clustal omega Web site (https://www.ebi.ac.uk/Tools/msa/clustalo/), which employs HHalign11 as an alignment mechanism, which is based on hidden Markov models (HMMs), in addition to the quick alignment that can only be done between a pair of sequences.^47^ Also, the phylogeny was performed on the Phylogeny Web site (https://www.phylogeny.fr/).^48,49^

### Structural Analysis

The PDBSum Web site (http://www.ebi.ac.uk/pdbsum/) was used to search for secondary structures and motifs, employing the PDB codes of proteins taken from UniProt. Once the motifs were identified, a comparison of the 3D structures of the proteins was carried out to identify the similarity of their structures. For such a task, only structures with a higher X-ray resolution were selected. Then, by using PyMOL’s (v. 2.5.0) align function, the A chains of the three selected enzymes were superimposed. PyMOL’s align function performs a multistep alignment of the aa sequence, followed by the aa structural alignment, thus obtaining the RMSD (root-mean-square deviation) according to eq 1.

$$\mathrm{RMSD}=\sqrt{\frac{1}{N}\sum^{N}(\alpha_{ix}-\beta_{ix})+(\alpha_{iy}-\beta_{iy})+(\alpha_{iz}-\beta_{iz})}$$ 1

where *N* corresponds to the total number of atoms in the system whose simple Euclidean distances in *x*, *y*, and *z* of the *i*th atom of the target structure α concerning the reference structure β are summed in a pairwise manner.

To make sure all alignments reached their minimum optimized aligned RMSD value, a maximum cycle of refinement of 200 steps was set, employing BLOSUM62 (Blocks Substitution Matrix for proteins with >62% of aa sequence similarity).^50^

### Mutation Studies

Once the structural comparison among the selected enzymes was obtained, we investigated the possible conformational changes caused by mutations in protein structures; for that, the DynaMut Web site (https://biosig.lab.uq.edu.au/dynamut/) was used to assess the probable mutations the PAI enzymes could undergo.

The DynaMut server performs a quick analysis of the protein stability in the face of a dynamic mutation, calculating the change in protein folding free energy (ΔΔ*G*) as a function of the thermodynamic state, meaning that hypothetically, the folding free energy of a mutant protein going to a wild-type protein must be equivalent to the negative of a wild-type protein going to a mutant protein.

In addition, DynaMut calculates the stability of the protein mutation by four other methods—normal-mode analysis (NMA) with Bio3D^51^ and ENCoM,^52^ site-directed mutator (SDM),^53^ mutation cutoff scanning matrix (mCSM),^54^ and DUET,^55^ allowing considerations about interactions of specific side chains of atoms.

### Docking and Dynamics

The chosen putative inhibitors extracted as natural compounds from *S. miltiorrhiza* were salvianolic acid B (SAcB), rosmarinic acid (RAc), caffeic acid (CAc), danshensu acid (DAc), tanshinone I (TAN), tanshinone IIA (TANIIA), dihydrotanshinone (DIHYDRO), cryptotanshinone (CRYPTO), and tanshinone IIA sulfate (SULTANIIA), as shown in Figure 9.

**Figure 9:**
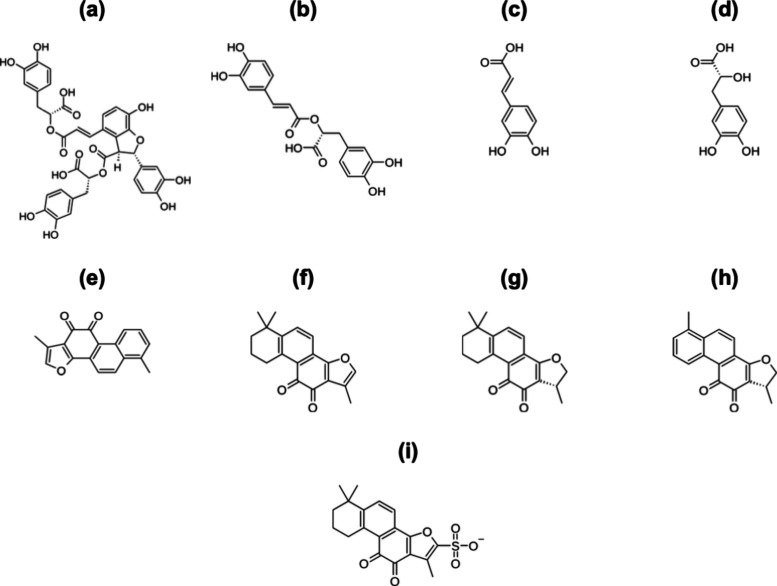
Molecular structures of (a) SAcB, (b) RAc, (c) CAc, (d) DAc, (e) TAN, (f) TANIIA, (g) CRYPTO, (h) DIHYDRO, and (i) SULTANIIA.

Docking analysis was conducted by taking into account a known binding site of PAI-1 and extending that known site to the other PAI enzymes. The 4G8O PDB structure is found with a known inhibitor bound to a specific site that was taken as a reference point for the location of the binding sites in the other PAI structures.

By aligning the 4G8O with the chosen models of PAI-1, PAI-2, and PAI-3, we ended up with a superposition that allowed us to determine which aa are close to the inhibitor for each PAI model within a distance range of 5 Å.

As the chosen protein structures presented missing 3D data, with only the aa sequence defined, we subjected all PAI structures to AlphaFold DB prediction^56,57^ in its default settings for the construction of models showing a complete 3D structure.

Once the models were created, we selected those with the lowest RMSD error, concerning the Cα backbone superposition between the model and the original structure. Also, as there is no hydrogen atom in the crystallographic data, we subjected the protein structures to the H++ (v. 4.0)^58–60^ server for adding missing hydrogens at pH = 7.4, considering an aqueous medium with salinity around 0.14 mol/L and permissivity of 80 ε to reproduce the blood environment condition.

All ligand and protein structures were prepared by employing obabel (v. 3.1.0)^61^ and taking into account the partial charges according to the eem2015ba^62^ model (Cheminf B3LYP/6-311G/AIM). Hence, Vina (v. 1.2.5)^63^ was employed for the docking campaign starting with 5000 seeds, with an exhaustiveness of 64, with 32 modes, and allowing an energy variation of 0.75 kcal/mol.

Finally, as the ligands were explored, we selected the best binding ligands (lowest energy found) for performing molecular dynamics (MDs) in each of the tested PAIs. The MDs were performed within a time range of 100 ns, with the Charmm36 force field,^64^ while CGenFF^65–67^ was used to construct the ligand topology files. All MDs were computed employing GROMACS.^68–75^

Once the simulation was accomplished, we employed the molecular mechanics/generalized Born surface area (MM/GBSA) scheme^76^ to estimate the binding free energy. The mathematical details of the MM/GBSA computation were presented elsewhere.^77–79^ The binding free energy (Δ*G*) was determined from the free energies of the PAI protein, the ligand, and the complex according to eq 2.^80^

$$\Delta G=G_{\mathrm{complex}}-(G_{\mathrm{ligand}}+G_{\mathrm{protein}})$$ 2

The free energy of each species (protein, ligand, and complex) was estimated using eq 3.

$$G=E_{\mathrm{vdW}}+E_{\mathrm{el}}+G_{\mathrm{GB}}+G_{\mathrm{Surf}}$$ 3

where *E*_vdW_ and *E*_el_ represent the van der Waals and electrostatic interaction energies, respectively. The polar solvation free energy (*G*_GB_) was estimated using the generalized Born equation, while the nonpolar solvation free energy (*G*_Surf_) was determined using eq 4.

$$G_{\mathrm{Surf}}=\gamma A+b$$ 4

with γ = 0.00542 kcal mol^–1^ Å^–2^ and *b* = 0.92 kcal mol^–1^. The symbol *A* denotes the solvent-accessible surface area (SASA). The GB model (igb = 2) developed by Onufriev et al.^76^ was used in this study. All of the simulation period was subjected to the computation of the binding free energy.

### Rationale for the Selection of Computational Tools and Databases

The computational tools and databases employed in this study were selected based on their robustness, reliability, and broad acceptance in the scientific community. Each was chosen to support specific structural and functional analyses with methodological precision:

**UniProt** was used to obtain amino acid sequences and functional annotations of plasminogen activator inhibitors (PAIs). It is a highly curated and widely recognized protein database, offering reliable and up-to-date information.

**PFam** was employed to identify conserved domains and protein families through hidden Markov models (HMMs), allowing for accurate functional inferences based on family-level classifications.

**Clustal Omega** was selected for multiple-sequence alignments due to its high alignment accuracy and computational efficiency. It is particularly suitable for phylogenetic and residue conservation analyses.

**Phylogeny.fr** was utilized for constructing phylogenetic trees as it integrates widely used tools such as MUSCLE, Gblocks, PhyML, and TreeDyn into an automated and reproducible pipeline for evolutionary studies.

**PDBSum** was used to analyze the secondary structures and structural motifs of the PAIs, providing detailed visualizations and structural summaries derived from high-resolution crystallographic data.

**PyMOL (v. 2.5.0)** served as the platform for structural alignment and RMSD calculations, due to its flexibility and high accuracy in handling and superimposing three-dimensional protein structures with high integration with python programming language, which simplifies important steps associated with computing surface area, bond angles and distances, and RMSD.

**AlphaFold DB** was adopted to model the missing regions in the crystallographic structures. AlphaFold is currently the leading tool for accurate structure prediction based on deep learning models.

**DynaMut** was employed to evaluate the structural effects of the point mutations on protein stability and flexibility. It combines several prediction methods (ENCoM, SDM, mCSM, and DUET), offering an integrated assessment of conformational impact.

**AutoDock Vina (v. 1.2.5)** was chosen as a free solution for molecular docking studies due to its computational efficiency for organic molecules, more accurate scoring functions when compared to AutoDockFF, and ability to explore diverse ligand conformations.

**H++** was used for protein protonation due to its efficiency, accuracy, reliability, and comprehensive output.

**LigPlot+** was chosen as a free tool due to its clear and intuitive visualization of the interactions between ligands and protein’s binding pocket.

**GROMACS** was chosen as a free tool for performing molecular dynamics simulations. It is a high-performance package widely used for biomolecular modeling, with compatibility for CHARMM36 and CGenFF force fields and efficient scaling on parallel systems.

**gmx_MM/PBSA** was chosen due to both being a tool fully compatible with GROMACS and its relevance for computing free binding energy, as well as thanks to its great parallel capability in computing heavy trajectories with low computational cost.
